# Parents’ perceptions of the safe environment for every kid (SEEK) model in the Swedish child health services

**DOI:** 10.1186/s12887-024-05064-8

**Published:** 2024-09-14

**Authors:** Marie Golsäter, Eva Randell, Maria Engström, Steven Lucas

**Affiliations:** 1 Child Research Group, School of Health and Welfare, Jönköping University, Child Health Service and Futurum - Academy for Health and Care, Region Jönköping County, Jönköping, Jönköping, Sweden; 2 Department of Social work, Uppsala University, Uppsala, Sweden; 3 Department of Women’s and Children’s Health, Uppsala University, Uppsala, Sweden

**Keywords:** Child Health Services, Health promotion, Parent, Psychosocial risk factors, The SEEK model

## Abstract

**Background:**

The Safe Environment for Every Kid (SEEK) model was developed to address psychosocial risk factors (financial worries, depressive symptoms, major parental stress, alcohol misuse and intimate partner violence) in the pediatric primary care setting but has not been evaluated from the parents’ perspective. To further investigate the usefulness of SEEK, it is important to explore how parents perceive the model.

**Objective:**

The aim of the present study was to explore parents’ perceptions of the SEEK model as a part of regular health visits in the Child Health Services in Sweden.

**Participants and setting:**

Eighteen parents (13 women and five men) in two Swedish counties participated in the study.

**Methods:**

Semi-structured telephone interviews were conducted, and the resulting data were analyzed using reflective thematic analysis.

**Results:**

Three themes were identified: *Acceptance and understanding of the SEEK model in the child health services*,* The questionnaire as a bridge to a dialogue*, and *Feeling trust in the system and the child health nurse’s professional competence.* Further, an overarching theme was created that encompassed a core meaning of all three themes; *SEEK provides a process-oriented framework to receive support in parenting with a focus on child health.*

**Conclusions:**

The study showed that parents express both acceptance and understanding of the SEEK model and they perceive that the model provides an avenue for repeated dialogues about the family’s situation during the child’s upbringing and an opportunity to access support if needed.

**Supplementary Information:**

The online version contains supplementary material available at 10.1186/s12887-024-05064-8.

## Introduction

Child and parent wellbeing are central to the optimal growth and development of each child according to his or her own potential [1]. Multiple and integrated systems in the child’s environment affect how families function, and in this context, psychosocial factors are key determinants of the child’s potential to thrive [2]. Common psychosocial factors in the home that may negatively affect child health and development outcomes and increase the risk of child maltreatment include financial strain, parental mental health problems, significant parental stress, substance abuse, and intimate partner violence (IPV) [3].

Pediatric primary care professionals see a number of hurdles to universally asking parents about psychosocial problems. These include time constraints, lack of knowledge about the available screening tools, insufficient training on how to address a positive screen, lack of knowledge on the resources available to refer to, and fear that the parent will find the questions uncomfortable or intrusive [4–7]. Survey studies of how parents perceive being asked about psychosocial problems during child health visits show that parents are mostly positive to discussing such issues with the child’s primary care provider [8, 9]. Most of these studies have focused on single issues such as screening for post-partum depression or substance use or abuse [10]. One study that evaluated parents’ experiences of multifaceted methods for identification of several psychosocial risk factors also shows a high degree of acceptability among most parents [11].

The Safe Environment for Every Kid (SEEK) model was developed to address psychosocial risk factors in the pediatric primary care setting [12]. The aim of the SEEK model is to help improve child health and development, strengthen families, and support parents by identifying common problems that parents may have, discussing what services parents may be willing to accept, and putting them in contact with the relevant resources. Randomized trials of the SEEK model have shown reductions in the use of harsh parenting, fewer reports to child protective services, and increased discussions about psychosocial issues with parents during child health visits in pediatric practices that employed SEEK [13, 14].

The Child Health Services (CHS) in Sweden is a part of primary care and aims to support children’s health and development. The CHS is offered free of charge to all children 0–5.

years of age and their parents. Uptake is nearly complete, with over 99% of children enrolled [15]. The CHS follows a national program with at least 16 age-specific visits, including two home visits; four of the 16 visits are team-based visits with a physician (GP or pediatrician) and a CHS nurse. The CHS focuses on health promotion, primary prevention, and early detection of health or developmental problems or risk factors in the child’s environment and referral to appropriate services [16]. Together, the focus on child and family health, ubiquitous coverage in the population, high level of trust among parents, many recurring visits, and a high level of continuity make the CHS an ideal environment to address psychosocial issues in order to promote children’s health [16].

The SEEK model has recently been introduced into the CHS context in several Swedish counties. The implementation of SEEK in a county has consisted of several steps. In the initial pilot phase, 3–5 CHCs in one or two municipalities used the model. Experiences from the pilot were then used in the subsequent expansion phases. Usually, two to three expansion phases took place before all units in a county had implemented SEEK. Today, the SEEK model is used in 11 (of 21) counties in Sweden. During the period of 2020–2023, a total of six counties had fully introduced the model, and an additional five counties were in the pilot or expansion phase.

The primary focus of the SEEK model is to prevent ill health and reduce the risk of child maltreatment, and the model provides a method for identifying children living in families with psychosocial risk factors [13, 14].

At five of the regular health visits when the child is 6–8 weeks, 10 months, 18 months, two and a half years, and four years of age, the parents are offered to fill in the Swedish version of the SEEK Parent Screening Questionnaire (PSQ), comprised of 17 questions including child safety, economic worries, parental depression, extreme parenting stress, alcohol misuse, and IPV. The parents’ answers are then used as a starting point for a dialogue concerning how any existing problems may affect the family and the child. Based on each family’s individual needs, further support by the CHS nurse or other services, e.g. a social worker, is offered according to the wishes of the parent [17].

Two studies have thus far examined the CHS nurses’ experiences of addressing psychosocial risk factors in the families they meet and found that the SEEK model provided a previously lacking structure that facilitated the identification of psychosocial risk factors. Further, the model made the issues approachable and supported the professionals in their sense of competence and security in addressing complex psychosocial issues [17, 18]. To further investigate the usefulness of the model, it was of vital importance to explore how parents’ perceived answering the PSQ and discussing psychosocial issues, which has not been previously studied. The aim of the present study was to explore parents’ perceptions of the SEEK model as a part of regular health visits in the CHS in Sweden.

## Methods

### Design

A qualitative explorative design [19] employing semi-structured interviews was used to obtain a detailed understanding of parents’ perceptions of the SEEK model as a part of regular health visits in two counties in Sweden. According to statistics Sweden (SCB), one of the counties has about 370,00 inhabitants divided into 13 municipalities with a variation from 6,900 to 147,000 inhabitants, and the second county has about 288,000 inhabitants divided into 15 municipalities varying from around 6,800 to 60,000 inhabitants. Both counties have a combination of urban and rural areas.

### Participants

A convenience sampling method was used to recruit parents to the study. At regular child health visits, parents at seven different CHS units were informed by their CHS nurse about the study, and parents who were interested in taking part received an information letter and were contacted by one of the researchers by telephone.

Eighteen parents (thirteen women and five men) whose children were enrolled in the national CHS program took part in telephone-based individual interviews. The last conducted interviews established aspects already existing in the data without adding new information, suggesting we had approached saturation.

The parents’ age ranged from 25 to 41 years (mean age 33), and all were born in Sweden. Altogether, the parents had 33 children aged two months to 11 years. In one family, both parents were interviewed, and in all other families, one of the parents participated. Most of the parents had been exposed to the SEEK model at more than one health visit.

### Data collection

The telephone interviews were conducted by three of the authors; ME conducted three interviews, MG conducted six, and ER conducted the remaining nine interviews. The interviewers had not previously met the parents and had not worked clinically with the SEEK model themselves. The 18 interviews were conducted between December 2021 and January 2022. The interviews lasted 15–42 min (median 23 min) and were audio-recorded and transcribed verbatim.

An interview guide was used to ensure that the central topics of interest were discussed. Each interview started with the question, “What are your thoughts about CHS nurses using a questionnaire to ask about various factors that can affect young children’s family environment?” Subsequent questions related to the following aspects found in the SEEK questionnaire: Child safety; smoking; economic worries; parental depression; stress; IPV and alcohol misuse. Questions were then posed about how the parent perceived the questions in the PSQ and the subsequent dialogue with the CHS nurse. Finally, questions were posed about how the parent’s responses and expressed needs were handled by the nurse.

### Data analysis

Reflexive thematic analysis (TA), according to Braun and Clarke [20], was used to analyze the interviews. In the initial phase, all transcripts were read through several times by MG and ER to become initially acquainted with the data. In the next step, data segments that were relevant for the aim of the study were highlighted in the coding process, and preliminary codes were developed and further discussed. The preliminary codes from the initial reading were compared between MG and ER to create a first common understanding. Through continuing revision of codes and reflection, the core understanding of the data was generated, and the codes were clustered to generate potential themes. A theme in reflexive TA is a pattern of shared meaning around an organizing concept [20]. Themes were further reviewed against codes and data. The analysis was developed from a semantic understanding to a more latent one in an ongoing reflexive discussion by MG and ER striving for a more refined interpretation of the parents’ perceptions of the SEEK model. In the later phases of the analysis process, all authors were involved in discussing the findings.

## Results

Based on the analysis of the parents’ experiences and thoughts, three themes were developed; *Acceptance and understanding of the SEEK model in the CHS*,* The questionnaire as a bridge to a dialogue*, and *Feeling trust in the system and the child health nurse’s professional competence.* Each theme consisted of several sub-themes. In addition, an overarching theme was created that encompassed a core meaning of all three themes: SEEK provides *a process-oriented framework to receive support in parenting with a focus on child health.* An overview of the overarching theme, themes, and sub-themes is presented in Table 1.

**Table 1:** Overarching theme, themes and sub-themes developed from interviews with parents exposed to the SEEK model

SEEK provides a process-oriented framework to receive support in parenting with a focus on child health
**1. Acceptance and understanding of the SEEK model in the CHS** *1.1 Relevant issues for the sake of all children* *1.2 Paying attention to the family’s life situation as a whole* *1.3 The CHS as a neutral arena for parenting support and health promotion*
**2. The questionnaire as a bridge to dialogue** *2.1 Structured questions provide specific answers* *2.2 The questionnaire as a starting point for discussion and reflection* *2.3 Recurrent questions provide repeated opportunities* **3. Feeling trust in the system and the nurse’s professional competence** *3.1 Safe and confident in receiving support from the CHS* *3.2 Trusting the nurse in the encounter* *3.3 Trust is created through several visits*

### SEEK provides a process-oriented framework to receive support in parenting with a focus on child health

Parents showed both acceptance and understanding of the SEEK model and were willing to answer questions about their psychosocial situation. The dialogue, based on the structured SEEK PSQ combined with the parents’ confidence in the CHS, enabled parents to get early support. According to the parents, confidence in the CHS and the professionals’ knowledge provided a basis for parents to take advantage of the support offered. Several processes were involved: the process of gaining confidence in the CHS, the process of child development in the early years, and the process of the family’s changing needs for support. Therefore, the SEEK model provided a process-oriented framework for parents to receive parenting support with a focus on the child’s health.

### Acceptance and understanding of the SEEK model in the CHS

The parents expressed that the questions dealt with relevant issues in the child’s best interest and that they focused attention on the family’s life situation as a whole. The CHS was perceived as a neutral arena for all parents and especially for those parents in need of support by following children and families through the crucial early years of the child’s life. Offering the SEEK model to all families normalized the method for the parents.

#### Relevant issues for the sake of all children

The parents expressed an understanding that universal application of the SEEK model in the CHS could support the entire parenting community. They described thinking that, by inviting every parent on several occasions to answer the questionnaire and discuss their situation, the parents’ sense of security would be strengthened and they would dare to express their needs. The parents commented that this was especially true for parents with several psychosocial risk factors, such as depression, violence, or alcohol abuse.

“The questions I think are quite relevant. Yes, I guess I don’t take it personally in any way, but I see it as the big picture that the CHS nurse needs to be informed about and can quickly see if there are gaps in parents’ knowledge and perhaps new parents in particular” (father, interview person 10).

The parents’ thoughts regarding the SEEK model included an overall sense of the importance of trying to make it easier for families with difficult life situations to get help. The parents expressed a sense of responsibility for all parents and children and especially, thanks to the support provided by the SEEK model, that the CHS were able to find parents and children in need of support. A mother explained why it was beneficial to ask questions:

“I think the CHS should ask questions. Because otherwise children might live in a home where things are not as they should be and the parents need help and support. So that things will be good for the child. And yes, it’s good if you can draw attention to such things so the parents can get that support” (mother, interview person 14).

#### Paying attention to the family’s life situation as a whole

Parents expressed that all questions in the questionnaire were relevant for parents with children aged 0–5 years as they concerned factors that may affect the wellbeing and safety of a family. According to the parents, the different issues that the questions covered helped to shed light on the family’s life situation as a whole, including the well-being of the child. The parents described that the questions were about many things in addition to the child’s health and development, including how the parents felt. The parents described many thoughts they had about how important it was that parents feel and function well.

As one mother put it, “I realized after these questions that there is so much more, it is absolutely mostly about how we as parents feel and our ability to take care of the child. So, I thought about how important it is that we also feel well, that we function” (mother, interview person 5).

Several parents highlighted that the questions regarding the family’s financial situation brought up an issue that may affect the entire family and that they had not thought about previously. They considered it important to be asked about possible financial worries. One mother commented, “I thought the questions were very good actually, I remember mainly reacting to that question about financial security, like, “They talk about things like this too, that’s great!” (mother, interview person 11).

Paying attention to the family’s life situation involved including fathers or partners as well as mothers: “I was offered an individual parent visit, and it was something I wanted to do because I felt being a parent was very hard in the beginning. I had very good support from home, but I felt that it was important to talk, and maybe not just for my sake, but to make it clear that men can also feel that it’s difficult” (father, interview person 10).

#### The CHS as a neutral arena for parenting support and health promotion

The CHS was considered to be a neutral place where parents felt confident that the services focused on promoting the child’s well-being. Even if the role of the CHS was not clear for the parents, they appreciated the CHS as an arena. One father said. “I don’t know exactly what kind of function the CHS has, but I suppose they have some idea of how the parents are, in case the children might be at risk, and can help. That’s what I think. No one has ever said that, but I have understood through the questions that they want to know if the parents are good parents, and I think that is very positive” (father, interview person 16).

The important role of prevention and support provided by the CHS was highlighted; “There is no one else at that early age, there is no school, so only the CHS has the opportunity to get a picture themselves, and it is also important to work preventively, to get parents to reflect, and that interaction is based on gentle pedagogy… which simply enables the parents’ own reflections on parenting” (mother, interview person 9).

Because the SEEK-questionnaire was offered to all parents they thought that it would make it easier for parents not to feel singled out or questioned. One mother explained how she experienced receiving the questionnaire: “She just showed me the questionnaire and said that this is something we always do, everybody gets this form, it’s not just you. It’s standard” (mother, interview person 12).

### The questionnaire as a bridge to a dialogue

The questionnaire was a starting point for discussion and reflections. The structured questions provided specific response alternatives, and the parents felt that being asked the same questions on several occasions provided opportunities for further reflection and consideration.

#### Structured questions provide specific answers

The parents described how the structured questionnaire opened the door to more in-depth reflections in contrast to more generally framed oral questions about the family’s well-being. One mother highlighted the universal approach: “She introduced it [the questionnaire] as something universal, something voluntary, and something important” (mother, interview person 7).

Parents described that broad questions about wellbeing, such as “how are things at home?”, encouraged responses that were general and less reflecting. The structured questions in the SEEK model facilitated for the parents to both reflect over and describe their situation in a dialogue with the nurse:

“If our nurse hadn’t asked these questions, I don’t think my partner would have talked to her about these things that she felt” (father, interview person 9).

Being asked as part of a universal routine also made it easier to talk about problems: “It’s probably too deeply hidden for many to tell. So, it’s easier when it happens on a routine basis, like you need to give an answer, then you might let it out. I think so, it’s always easier if someone actively asks. So, that’s great. If you ask, you get an answer. If you get a question, it makes it easier to tell” (mother, interview person 1).

#### The questionnaire as a starting point for discussion and reflection

The main task of the questionnaire, according to the parents, was as a starting point for dialogues, not just to get answers without further follow-up. “It is a way to get control of the conversation. So, it’s great that the questionnaire exists, because I think about this conversation that we had the last time, the form itself served as a starting point for a broader conversation, where you kind of pick up different parts and feel what need there is” (father, interview person 4). The parents described how the questions opened up to reflection about factors that affected the family in ways they previously had not reflected on or when they needed help: “I think it was a good conversation because that’s when this depression issue came up. So that was sort of what we discussed. And I did get help” (mother, interview person 14).

Parents also described that the questionnaire clarified their situation both in the dialogue with the CHS nurse and in a longer perspective after the visit when the parents continued to reflect on their situation both individually and in further conversations with their partner. The parents described that in some cases it might be difficult to answer the questions honestly, for example when a parent hasn’t thought about the connection between their problems and consequences for the child’s well-being or when a parent was afraid of being questioned or criticized by the nurse.

#### Recurrent questions provide repeated opportunities

The parents highlighted how circumstances that may affect a family during the child’s early years of life may vary over time and therefore appreciated that the SEEK model repeatedly follows the families during the crucial years of early childhood. The changing needs of the parents and their children during the early years were also emphasized. Answering the SEEK questionnaire at several CHC visits during the child’s upbringing made it easier for parents to express their needs and receive support in the long run. As one father explained:

“The questions come up again, and so do the themes… exactly. It’s great, I thought that the nurses get feedback all the time. And also… some kind of reminder that a lot can happen during these years as time goes by. New sides of reality and new sides of yourself and your family” (father, interview person 4).

The parents described that it was important to explore their needs on several occasions during their child’s first years of life. The parents conveyed that they saw the child’s first five years as a process of development for both the child and the family and that the need for support could vary depending on the child’s developmental phase and the family’s situation.

### Feeling trust in the system and the nurse’s professional competence

Feeling safe and confident with the CHS was a prerequisite for getting help, and many parents expected to receive adequate support and assistance. The nurse’s professional and relational competence was important in the encounter, and trust was often created through several encounters.

#### Safe and confident in receiving support from the CHS

The parents expressed a feeling of trust in the CHS as a part of the social welfare system in Sweden and in the competence of the nurses. They described that the SEEK model was perceived as an accepted and well-functioning part of the CHS. Based on their confidence in the CHS, the parents described an expectation of being provided with help and support when they expressed their worries through the SEEK questionnaire and the following discussion with the nurse. One parent described having difficulties with their child: “Our daughter has a lot of emotions, and that was something that my partner brought up with our nurse. She helped us get in touch with someone to talk to about the whole situation. When the nurse felt that she didn’t have the skills herself, she referred us on to someone who did. Fantastic.” (father, interview person 16). The parents described that the CHS nurse’s caring relationship facilitated for them to express needs and problems that arose in the family at later health visits. A parent highlighted a feeling of security because the nurse showed that she was available: “When I talked to her [the nurse] about how I was having a hard time, she kept offering contact with family therapists and so on. So, I always felt that she was there if I needed help” (mother, interview person 11).

#### Trusting the nurse in the encounter

Parents described that they felt safe and confident and were able to bring up difficult problems when they felt trust in the nurse’s competence and ability to care, and that this increased over time.

A father described how the mother of their child felt during a visit: “It was a neutral person who asked the questions, someone she felt she trusted (father, interview person 9).

Trusting the nurse was also a path to gathering the courage to talk about more difficult problems: “If I feel safe to tell my nurse how I’m feeling, it’s easier, it’s a doorway to talk about other things if I feel bad… So, stress and depression feel like the entrance, if I dare to talk about that, then I might dare to talk about threats and violence” (mother, interview person 7).

#### Trust is created through several visits

The parents described that the frequent continuous encounters with the nurse during the child’s first year provided the conditions for developing and deepening a caring relationship if the nurse treated the parent with trust.

“The relationship is very important. If I had a problem, I wouldn’t open up if I met a temporary nurse. I would have waited until my regular nurse was in place, because you have a relationship, I think the relationship is very important, so you have the courage to say that things are not going well. (mother, interview person 2)

The parents described that, in the context of this caring relationship with the nurse, they expected to be met with mutual understanding, based on their own thoughts and experiences and knowledge of their own situation. Trust and confidence grew over the course of years:

“We have confidence in our nurse because there are so many different areas where she shows great competence, and we have gained more and more confidence over several years… Her experience and her personality fit well with ours, and the way she offers information is more like asking, “Can I give you information about… what the latest studies say about… different things” (father, interview person 16).

The relational trust created a feeling of “knowing each other”, as a father explained:

“Confidence building. You’ve been able to talk… Yes, I think she’s been good from the start, and it’s worked out great. It has felt very secure. You feel that you almost know each other, even if you only meet once a year or every six months” (father, interview person 10).

## Discussion

This study aimed to explore parents’ perceptions of the SEEK model at health visits in the CHS. The overarching theme *SEEK provides a process-oriented framework to receive support in parenting with a focus on child health* illustrates the parents’ favorable opinions of the SEEK model as a way to get support through the families’ years of contact with the CHS.

The results showed that parents accept the model as part of the CHS’s approach to health promotion. Parents showed both acceptance and understanding of the SEEK model and showed a collective responsibility for all children. Through a normalizing, health-promoting approach addressed to all parents, psychosocial risk factors could be expressed by the parents and identified by the CHS nurse. The parents highlighted the importance of regularly asking questions to identify families in need, even if they themselves did not require support. The CHS was felt to be a neutral arena that made it easier for parents to get early support based on their needs. Further, the parents described that the model enabled the nurse to capture the family’s changing situation and needs over time.

### Acceptance and understanding of the SEEK model in the CHS

The parents in the present study described good general acceptance and understanding of the SEEK model as a natural part of the CHS’s overall health-promoting work focusing on relevant aspects of the child’s and the parents’ life situation. The model reinforced and enhanced the CHS nurses’ parental supportive work and could strengthen a child’s health and development. This description is in accordance with the overall aim of the national CHS program in Sweden [16].

The parents thought that the SEEK model facilitated discussions about sensitive topics concerning the child and the parents’ situation by using the same questions for all parents. In another study [21], mothers also expressed the value of using the same questions with all parents regarding questions about intimate partner violence in the CHS context. The mothers highlighted the importance of offering the same questions to everyone to avoid the feeling of being singled out [21], which was also evident in the present study. The parents’ thoughts were also in line with results from a study among Swedish CHS nurses, who found that the SEEK model fit well into the CHS program and that it was valuable in identifying families in need of extra support [17]. In the United States, the SEEK model was also described as useful in helping health professionals reach families with psychosocial risk factors [22]. In a recent study, parents expressed a high degree of acceptability for universal screening for psychosocial risk factors among all parents in the context of well-child visits, findings that are in line with those of our study [11].

Selvaraj et al. [23] highlighted the need for regular screening for risk factors to enable parents to talk about their situation and needs, as readiness to disclose problems may come after several encounters. Parents also described the SEEK model as a way to open up valuable conversations about topics that are not usually discussed with parents [22]. As the family’s situation and needs often change over the course of the child’s first years of life, the parents in the present study and the parents in the study by Selvaraj et al. [23] described the advantage of reiterating screening instruments at different time points. This indicates the need for repeated universal screening during childhood. As the national CHS program in Sweden is based on many age-related health visits, the SEEK model can be used several times within the framework of the program [16].

### The questionnaire as a bridge to a dialogue

The SEEK model applies a holistic perspective, where the child and family are seen in a common context. The parents understood that the questions were provided for the sake of all children in the municipality, especially those children in need of support. The questionnaire, offered to all parents, normalized the procedure and was perceived as a good starting point for an in-depth conversation. This is well in line with the contextual dimension of health and wellbeing and a socioecological understanding that focuses on the child in different contexts linking the individual and their surrounding environment [24, 25].

The parents felt that the questionnaire opened up for dialogue where it was possible to discuss family problems and concerns regarding the child. Parents explained that they answered because the questions were asked, and some explained that they probably would not have brought up the issue if they hadn’t been asked directly. Therefore, this study suggests that asking direct questions provides answers that likely would not have been offered spontaneously. Continuous health dialogues are in accordance with the aims of the CHS [16], and such dialogues provide guidance and understanding as well as space for children’s and parents’ involvement [26]. In one study [27], CHS nurses highlighted that using a questionnaire can lead to more responses regarding sensitive issues compared to asking verbal questions in a conversational form. Simply asking such questions demonstrates to the parent that the conversation is essential and that it is permissible to talk about the issues [27].

The SEEK model was a way to include aspects such as intimate partner violence, family finances, and alcohol use that the CHS nurses previously did not usually discuss with parents. Modern health promotion work includes the whole family, which places increasingly complex demands on the nurse’s competence to communicate about and meet different needs. It is of vital importance to individualize the support and advice offered according to the specific needs of each parent and child. Fathers were offered individual “partner visits” with the nurse, which they found positive. Paternal mental health is intimately associated with child mental health and developmental outcomes from birth and throughout the child’s lifespan [28] underscoring the importance of identifying fathers in need of help.

Parents reported that the CHS nurse offered referrals to other professionals, such as a psychologist or other mental health professional, based on factors that emerged in the SEEK- based dialogue. Thus, connecting parents with other resources that can support them was a result of the SEEK model and all professionals were seen as a pool of resources. Our findings showed that the positive attitude and competence of the CHS nurse were crucial in creating a supportive climate and initiating dialogues with the parents, even about difficult and demanding issues.

### Feeling trust in the system and the nurse’s professional competence

The study showed that an important prerequisite for the CHS to function as a supportive environment was that parents felt trust in the CHS nurse and the CHS as an arena. According to the parents’ experiences, the CHS was an important and neutral setting for health promotion and family support. Feeling trust in the nurse and the system was an important cornerstone. Trust was built through a process of dialogue over the course of several visits during the child’s early years. Trust enabled the parents to express needs and receive support.

Several parents highlighted that there were many challenging changes and demands during their child’s early years. Being able to meet and support families with complex needs placed demands on the nurse’s competence. Studies have identified the importance of early life experiences for people’s health throughout the life course, and studies have established the link between adverse childhood experiences (ACE) and ill-health in adulthood [29, 30]. ACEs include harms that affect children directly, such as neglect, and indirectly through their home environments, such as household financial problems and parental substance abuse. Children with ACEs suffer from more physical and mental health problems as adults compared to those without ACEs [31]. Counteracting childhood stressors is thus crucial to finding ways to counteract later ill-health. The parents in our study felt that the CHS nurse could strengthen their parenting skills by providing adequate knowledge and individualized support to increase psychological well-being. One study supporting parents during the first two years found that parents prefer support that is tailored to their personal needs and practices based on a trusting relationship between healthcare professionals and parents [32]. The concept of trust is in accordance with an empowerment approach, which is about promoting individuals’ health and quality of life through support that strengthens the individual’s control over factors that affect their family life [33, 34]. How the parents perceived the competence and skills of the CHS nurse in this study and the extent to which they trusted the nurse to act and support them, influenced what they brought up in the encounter. According to Bidmead et al. [35], nurses need to make the parent feel safe, highlighting kindness, reliability, empathy, openness, and honesty as essential qualities in the meeting to help the parent to open up and be honest. The present study showed that through trust and continuity, the CHS nurse was able to raise awareness of issues that affect children, and many parents themselves were able to reflect on the consequences and change their actions for the benefit of the child.

## Methodological considerations

This study’s results should be reflected in relation to its limitations. Parents visiting the CHS during a time period of approximately 6 weeks were informed about the study by the CHS nurse. Those who were willing to participate in the study gave their consent to be contacted by researchers.

We lack data on how many parents were asked and how many refused, but all parents who were contacted agreed to participate. The data collection was limited to parents living in two of Sweden’s 21 counties, and all parents were born in Sweden. The fact that we did not reach immigrant families is a disadvantage. Earlier studies show that immigrant parents to a lower degree feel trust in the health care system [36]. Thus, further studies including immigrant parents are needed. However, both mothers and fathers participated, and both well-functioning parents and parents in need of extra support from the CHS took part in the interviews, which could be seen as a strength as we to some extent also gathered experiences from parents in need of extra support.

Potential sources of bias include the recruitment process, which may have led to inclusion of the parents who were mostly positive to the SEEK model and the CHS as a whole, while more critical parents may have declined participation. We cannot assess the extent to which this may have affected the results, but the possibility must be acknowledged.

The three interviewers used a semi-structured interview guide to ensure that the interviews were conducted in a similar way. The data gathered were rich in describing the parents’ various experiences and thoughts about taking part in the SEEK model during their regular visits to the CHS. To ensure the trustworthiness of the data analyses, two of the authors (MG and ER) performed the analysis in an ongoing reflexive discussion as described by Braun & Clarke 2022. Quotes from the parents’ descriptions were used to further describe the content of the themes. The authors who conducted the analysis had no personal experience of the SEEK-based conversations and thus no preconceptions, which may have contributed to a more open approach in the data analysis. The research team consisted of two registered specialist nurses, one pediatrician and one social worker, contributing to interdisciplinary perspectives in the analysis. All authors collaborated in the analysis and were involved in negotiating the findings.

## Conclusions

The study showed that parents are both accepting and understanding of the SEEK model. They perceive that the model provided a way to initiate recurring opportunities for dialogue about the family’s situation and to receive support if needed throughout the child’s first years of life.

### Implications for clinical practice

As part of the CHS’s efforts to promote health, the parents demonstrated acceptance and comprehension of the SEEK model and its aims. The parents’ comments made it clear that use of the SEEK model in the CHS provides an ideal opportunity to address psychosocial issues in order to improve clinical practice and promote children’s health.

## Electronic supplementary material

Below is the link to the electronic supplementary material.

Supplementary Material 1

## Data Availability

Availability of Data and materials. The dataset supporting the conclusion of this article is not publicly available due to ethical reasons. The data are available from the corresponding author upon reasonable request.

## References

[1] Newland LA. Supportive family contexts: promoting child well-being and resilience. Early Child Dev Care. 2014;184(9–10):1336–46. 10.1080/03004430.2013.875543

[2] Walker SP, Wachs TD, Grantham-McGregor S, Black MM, Nelson CA, Huffman SL, et al. Inequality in early childhood: risk and protective factors for early child development. Lancet. 2011;378(9799):1325–38. 21944375 10.1016/S0140-6736(11)60555-2

[3] Dubowitz H, Finkelhor D, Zolotor A, Kleven J, Davis N. Addressing adverse childhood experiences in Primary Care: challenges and considerations. Pediatrics. 2022;149(4). 10.1542/peds.2021-052641 PMC9405315 35362065

[4] Szilagyi M, Kerker BD, Storfer-Isser A, Stein RE, Garner A, O’Connor KG, et al. Factors associated with whether pediatricians inquire about parents’ adverse childhood experiences. Acad Pediatr. 2016;16(7):668–75. 27157045 10.1016/j.acap.2016.04.013 PMC5563967

[5] Hornor G. Child maltreatment: Screening and Anticipatory Guidance. J Pediatr Health Care. 2013;27(4):242–50. 23791118 10.1016/j.pedhc.2013.02.001

[6] Paavilainen E, Helminen M, Flinck A, Lehtomäki L. How Public Health Nurses Identify and Intervene in child maltreatment based on the National Clinical Guideline. Nurs Res Pract. 2014;2014:425460. 25505986 10.1155/2014/425460 PMC4253703

[7] Trowbridge MJ, Sege RD, Olson L, O’Connor K, Flaherty E, Spivak H. Intentional Injury Management and Prevention in Pediatric Practice: results from 1998 and 2003 American Academy of Pediatrics periodic surveys. Pediatrics. 2005;116(4):996–1000. 16199714 10.1542/peds.2005-0618

[8] Hart CN, Kelleher KJ, Drotar D, Scholle SH. Parent–provider communication and parental satisfaction with care of children with psychosocial problems. Patient Educ Couns. 2007;68(2):179–85. 17643912 10.1016/j.pec.2007.06.003 PMC2099312

[9] Hayutin LG, Reed-Knight B, Blount RL, Lewis J, McCormick ML. Increasing parent–pediatrician communication about children’s psychosocial problems. J Pediatr Psychol. 2009;34(10):1155–64. 19270030 10.1093/jpepsy/jsp012

[10] Matson PA, Bakhai N, Solomon BS, Flessa S, Ramos J, Hammond CJ, et al. Understanding caregiver acceptance of screening for family substance use in pediatric clinics serving economically disadvantaged children. Substance Abuse. 2022;43(1):282–8. 34214411 10.1080/08897077.2021.1941510 PMC9901192

[11] Selvaraj K, Ruiz MJ, Aschkenasy J, Chang JD, Heard A, Minier M et al. Screening for Toxic Stress Risk Factors at Well-Child Visits: The Addressing Social Key Questions for Health Study. The Journal of pediatrics. 2019;205:244-9.e4. 10.1016/j.jpeds.2018.09.004 30297291

[12] Lane WG, Dubowitz H. Social determinants of health, personalized medicine, and child maltreatment. Pediatr Res. 2021;89(2):368–76. 33288877 10.1038/s41390-020-01290-9

[13] Dubowitz H, Feigelman S, Lane W, Kim J. Pediatric Primary Care to help prevent child maltreatment: the safe environment for every kid (SEEK) model. Pediatrics. 2009;123(3):858–64. 19255014 10.1542/peds.2008-1376

[14] Dubowitz H, Lane WG, Semiatin JN, Magder LS. The SEEK Model of Pediatric Primary Care: can Child Maltreatment be prevented in a low-risk Population? Acad Pediatr. 2012;12(4):259–68. 22658954 10.1016/j.acap.2012.03.005 PMC5482714

[15] Wettergren B, Blennow M, Hjern A, Söder O, Ludvigsson JF. Child Health Systems in Sweden. J Pediatr. 2016;177s:S187–202. 27666267 10.1016/j.jpeds.2016.04.055

[16] National Board of Health Welfare. Vägledning för barnhälsovården. Socialstyrelsen Stockholm; 2014.

[17] Engström M, Randell E, Lucas S. Child health nurses’ experiences of using the safe environment for every kid (SEEK) model or current standard practice in the Swedish child health services to address psychosocial risk factors in families with young children – A mixed-methods study. Child Abuse Negl. 2022;132:105820. 35932659 10.1016/j.chiabu.2022.105820

[18] Engström M, Hiltunen J, Wallby T, Lucas S. Child Health nurses’ experiences of addressing psychosocial risk factors with the families they meet. Acta Paediatr. 2021;110(2):574–83. 32716528 10.1111/apa.15492 PMC7891612

[19] Polit DF, Beck CT. Nursing research: generating and assessing evidence for nursing practice2012.

[20] Braun V, Clarke V. Thematic analysis: a practical guide. Los Angeles: SAGE; 2022.

[21] Almqvist K, Källström Å, Appell P, Anderzen-Carlsson A. Mothers’ opinions on being asked about exposure to intimate partner violence in child healthcare centres in Sweden. J Child Health Care. 2018;22(2):228–37. 29334792 10.1177/1367493517753081

[22] Eismann EA, Theuerling J, Maguire S, Hente EA, Shapiro RA. Integration of the safe environment for every kid (SEEK) model across primary care settings. Clin Pediatr. 2019;58(2):166–76. 10.1177/0009922818809481 30371116

[23] Selvaraj K, Korpics J, Osta AD, Hirshfield LE, Crowley-Matoka M, Bayldon BW. Parent perspectives on adverse childhood experiences & Unmet Social needs screening in the Medical Home: a qualitative study. Acad Pediatr. 2022;22(8):1309–17. 36007805 10.1016/j.acap.2022.08.002

[24] Bronfenbrenner U, Ceci SJ. Nature-nurture reconceptualized in developmental perspective: a bioecological model. Psychol Rev. 1994;101(4):568–86. 7984707 10.1037/0033-295X.101.4.568

[25] Bronfenbrenner U. Ecology of the family as a context for human development: research perspectives. Dev Psychol. 1986;22(6):723. 10.1037/0012-1649.22.6.723

[26] Håkansson L, Derwig M, Olander E. Parents’ experiences of a health dialogue in the child health services: a qualitative study. BMC Health Serv Res. 2019;19(1):774. 31666057 10.1186/s12913-019-4550-y PMC6820984

[27] Anderzen-Carlsson A, Gillå C, Lind M, Almqvist K, Lindgren Fändriks A, Källström Å. Child healthcare nurses’ experiences of asking new mothers about intimate partner violence. J Clin Nurs. 2018;27(13–14):2752–62. 29274181 10.1111/jocn.14242

[28] Fisher SD, Cobo J, Figueiredo B, Fletcher R, Garfield CF, Hanley J, et al. Expanding the international conversation with fathers’ mental health: toward an era of inclusion in perinatal research and practice. Arch Women Ment Health. 2021;24(5):841–8. 10.1007/s00737-021-01171-y 34431009

[29] Humphrey N, Wigelsworth M. Making the case for universal school-based mental health screening. Emotional Behav Difficulties. 2016;21(1):22–42. 10.1080/13632752.2015.1120051

[30] Bellis MA, Hughes K, Ford K, Rodriguez GR, Sethi D, Passmore J. Life course health consequences and associated annual costs of adverse childhood experiences across Europe and North America: a systematic review and meta-analysis. Lancet Public Health. 2019;4(10):e517–28. 31492648 10.1016/S2468-2667(19)30145-8 PMC7098477

[31] Hughes K, Bellis MA, Hardcastle KA, Sethi D, Butchart A, Mikton C, et al. The effect of multiple adverse childhood experiences on health: a systematic review and meta-analysis. Lancet Public Health. 2017;2(8):e356–66. 29253477 10.1016/S2468-2667(17)30118-4

[32] Boelsma F, Bektas G, Wesdorp CL, Seidell JC, Dijkstra SC. The perspectives of parents and healthcare professionals towards parental needs and support from healthcare professionals during the first two years of children’s lives. Int J Qualitative Stud Health Well-being. 2021;16(1):1966874. 10.1080/17482631.2021.1966874 PMC8405107 34435540

[33] Franzén C, Nilsson E-L, Norberg JR, Peterson T. Trust as an analytical concept for the study of welfare programmes to reduce child health disparities: the case of a Swedish postnatal home visiting programme. Child Youth Serv Rev. 2020;118:105472. 10.1016/j.childyouth.2020.105472

[34] Tengland P-A, Empowerment. A conceptual discussion. Health Care Anal. 2008;16(2):77–96. 17985247 10.1007/s10728-007-0067-3

[35] Bidmead C, Cowley S, Grocott P. The parental contribution to the parent/health visitor relationship. J Health Visiting. 2016;4(1):48–55. 10.12968/johv.2016.4.1.48

[36] Berlin A, Törnkvist L, Hylander I. Watchfully checking rapport with the Primary Child Health Care nurses - a theoretical model from the perspective of parents of foreign origin. BMC Nurs. 2010;9:14. 20646287 10.1186/1472-6955-9-14 PMC2918611

