# Supplementary material for: Parents’ perceptions of the safe environment for every kid (SEEK) model in the Swedish child health services
Source: BMC Pediatr. 2024 Sep 14;24:581. doi: 10.1186/s12887-024-05064-8 (PMC11401304; doi:10.1186/s12887-024-05064-8)
Supplement: Supplementary file 1 — Supplementary Material 1 [file 12887_2024_5064_MOESM1_ESM.docx]

*Supplement 1. Interview questions*

**Table 1. Questions included in the interview guide**

| Which aspects do you think are most important to ask about at the CHC?  How did the CHS nurse introduce the questionnaire to you?  How was it for you to answer the questionnaire the first time?  How was it for you to answer the questionnaire at the follow-up visits?  Was any question area easy/difficult/challenging to answer?  How did you and the CHS nurse discuss your answers in the questionnaire?  How did you experience the CHS nurse's competence in discussing the answers?  Did you have the opportunity to discuss what you wanted with the CHS nurse based on the questionnaire?  Were you offered follow-up/other support based on what emerged in the questionnaire or discussion? If yes, what do you think of the support that was offered?  What is the importance of your relationship with the nurse for answering the questionnaire and for the discussion?  What makes it easier to tell if you, as a parent, need help and support?  Would you talk about it if you need help and support?  How were you affected by the questions and the discussion - did you take ideas home with you?  Did you make any changes after the questions and discussion?  What could help make the health visit work best when the questionnaire is used?  Is there anything else you want to tell us? |
| --- |
